# Reporting The Effects of Exposure to Monosodium Glutamate on The Regulatory Peptides of The Hypothalamic-Pituitary-Gonadal Axis

**DOI:** 10.22074/IJFS.2021.522615.1072

**Published:** 2021-10-16

**Authors:** Muhammad Haddad, Rafat Esmail, Homayoun Khazali

**Affiliations:** 1 Department of Animal Sciences and Marine Biology, Faculty of Life Sciences and Biotechnology, Shahid Beheshti University, Tehran, Iran; 2 Department of Food Sciences, Faculty of Agriculture, Damascus University, Damascus, Syria

**Keywords:** Hormones, Neuropeptides, Neurotrophic Factors, Reproduction, Sodium Glutamate

## Abstract

Monosodium glutamate (MSG) is a flavour enhancer that is used as a food additive (E621) in many parts of the world, especially in East Asian countries. However, in recent studies, it has been used as a neurotoxin because MSG is re- ported to cause neural degeneration in the hypothalamic arcuate of neonatal animals. The results of several studies show the negative effects of MSG injections on different parts of the hypothalamic-pituitary-gonadal (HPG) axis, in addition to its ability to inhibit secretion many reproductive neuropeptides, neurotrophic factors, and hormones, all of which play vital roles in the regulation of reproductive function. Oral administration or injection of large quantities of MSG into newborn animals results in a decrease in or overabundance of the production of many regulatory peptides of the male and female reproductive systems. In this review, we summarize the results of the most important studies that have examined the effect of oral consumption or injection of MSG on regulatory peptides of the HPG axis.

## Introduction

Monosodium glutamate (MSG) is a common food additive for many foods from different countries, especially in East Asia. It has a special taste, umami, and is well-known in many countries by the name “savory” or “China salt”. MSG is added as a flavour developing agent (E621) in the form of hydrolysed protein or as purified monosodium salt (1).

The growing use of MSG in processed foods is welldocumented. It has been reported that more MSG is used in Europe (0.3-0.5 g/day) compared to Asia (1.2- 1.7 g/day) (2), and the daily acceptable intake of MSG is proposed to be 30 mg/kg/body weight (BW)/day (3). Despite reports that MSG is safe for consumption (4, 5), the results of several studies show its potential toxicity. Excessive MSG consumption may exacerbate asthma (6) and migraine headaches are related to glutamate (7). Furthermore, the reported correlation between subcutaneous injections of MSG and increases in mRNA expression of interleukin-6 (IL-6), tumour necrosis factor-alpha (TNFα), and resistin, in addition to peroxisome proliferator-activated receptors alpha and gamma (PPARα and γ) and liver transaminases confirms its negative inflammatory and metabolic effects (8, 9). The neurotoxic effects of MSG include destruction of cells in the hypothalamic arcuate nucleus and surrounding areas, which might lead to obesity (10).

here are many indications of the toxic effects of MSG on the male and female reproductive systems. The presence of hypogonadism in MSG-injected mice was corrected by injections of physiological concentrations of oestradiol (11). Moreover, the plasma concentration of inhibin B decreased significantly in MSG-treated male rats (12). Oral gavage of MSG to pregnant mice penetrated the placental barrier and reached foetal tissues. The level of MSG in the foetal brain was twice as high as the maternal brain, and this increase in MSG concentration in the brain reflected negatively on motor tests performed on the newborn mice (13). Moreover, nutrition supplemented with MSG for 40 days caused cytoplasmic vacuolations, swollen mitochondria, and shrunken nuclei in spermatogenic, Sertoli, and Leydig cells, in addition to defects in the tubular basement membrane, damaged germ cells and seminiferous tubules, decreased diameter and height of the lining of the epithelium, and disorders in spermatogenic cells in male albino rats (14). On the other hand, in female virgin rats, it was observed that oral gavage for 30 to 40 days caused significant increases in the duration of the diestrus phase; diestrus index; numbers of primary and primordial follicles; size of the Graafian follicle; and a decrease in the duration of the proestrus, estrus, and metestrus phases and size of the corpus luteum (15).

The mechanism of MSG-induced damage, regardless of the organ or cell type, is explained by the induction of oxidative stress (16). In this phenomenon, the levels of reactive oxygen species increase within the cell, which leads to damages to cell proteins, lipids, polysaccharides, and nucleic acids, and results in deficiencies in many cellular activities (17). This increase in oxidative stress in MSG-treated animals was inferred by an increase in enzymatic activity of superoxide dismutase (SOD), glutathione-s-transferase (GST), and catalase in the livers of the treated rats (18). MSG induces apoptosis via lipid peroxidation (LPO) in the arcuate nucleus, hypothalamus, and other circumventricular organs (19). In addition, this oxidative stress drives cells to apoptosis in the rat thymocytes by reducing Bcl-2 expression (20); however, this is accompanied by an increase in intracellular calcium, which triggers a cascade of enzymatic activities and subsequent apoptosis (21) in addition to activation of calcium-dependent protease, calpain and apoptosis-inducing factor (AIF) (22). Remarkably, this toxicity can be reversed after the use of antioxidants (14).

Numerous reports of conflicting results exist about the toxic effects and safety of MSG on the reproductive system, the nervous system, and regulating glands. Therefore, it is important to shed light on the effect of MSG on the production of sex hormones and peptides that regulate the HPG axis to determine its effect on reproductive function. This review article examines studies that assessed the effects of various routes of administration (oral, subcutaneous, intraperitoneal) of MSG on the production of regulatory peptides of the hypothalamic-pituitary-gonadal (HPG) axis.

### Monosodium glutamate and the hypothalamicpituitary-gonadal axis regulatory peptides

#### Monosodium glutamate and reproductive neuropeptides

Kisspeptin is a critical neuropeptide in mammalian reproductive function because it controls the secretion of gonadotropin-releasing hormone (GnRH) (23). The results of a study indicated that MSG plays an indirect catalytic role for luteinizing hormone (LH) secretion. On the one hand, MSG has a positive role in stimulating kisspeptin neurons (24). On the other hand, the results of recent studies have shown involvement of neurokinin B in regulating the pituitary-gonadal axis (25). Immunotechnology techniques revealed less immunoreactivity for both somata and fibers of neurokinin B-producing neurons within the arcuate nucleus in the neonatal MSG-injected animals compared to control animals (26). Neuropeptide Y (NPY) plays an important role in reproduction because it affects kisspeptin/neurokinin B/dynorphin secreting neurons and the GnRH pathway (27). In general, numerous study results show decreases in hypothalamic NPY levels in MSG-injected animals (28). In other words, levels of NPY in the mediobasal and mediodorsal hypothalamus reduced significantly after neonatal rats were injected with MSG; however, at the same time, its Y1 and Y5 NPY receptors up-regulated (29).

In contrast, expression levels of NPY increased significantly in both the hypothalamus and the pituitary gland in the MSG-treated rats (30). Another study mentioned that treatment of mice with MSG arrested expression of NPY mRNA in the arcuate nucleus and reduced pro-opiomelanocortin (POMC) mRNA in the hypothalamus (31). POMC is a precursor polypeptide that expresses within the hypothalamus, pituitary glands, and brainstem. It is cleaved to various important neuroendocrine peptide derivatives like melanocyte-stimulating hormones (MSHs), adrenocorticotropic hormone (ACTH), and others (32). At the same time, an increase in its production has a negative effect on reproduction; studies have indicated that neonatal MSG-injected rats evoked an increase in POMC expression accompanied by an increase in ACTH expression in the pituitary gland, thus, the appearance of a state of stress that has a negative impact on reproduction (33). Subcutaneous injection of MSG (4 g/kg bw) in rats depleted hypothalamic pro-opiomelanocorticotropin-derived peptides (34).

Galanin, which is produced with its receptors GALR1, GALR2, and GALR3 in the hypothalamus, pituitary and different parts of the male and female reproductive systems and galanin-like peptide, which is a hypothalamic neuropeptide that binds to galanin receptors, are two peptides that play an important role in regulation of metabolism and reproductive function (35). Galanin-like immunoreactivity in the neonatal rats that received subcutaneous injections of MSG (4 g/kg bw) had significant reductions in the median eminence (ME), medial basal hypothalamus, and septal and preoptic regions (36). In addition, immunoreactivity of galanin was completely lost in the arcuate nucleus neurons of female rats that received subcutaneous injections (4 mg/g bw) of MSG (37). Vasoactive intestinal polypeptide (VIP) is a member of a family of neuropeptides and endocrine peptides that have an important role in the control of testosterone levels and testes aging (38), and in the female reproductive system (39). Although subcutaneous treatment of neonatal male Wistar rats with MSG caused an increase of major axes and somatic area of VIP neurons in the suprachiasmatic nucleus (SCN), there was a significant decrease in the VIP-immunoreactive neuronal density (40). This VIP-immunoreactive in SCN increased in another study (41). Agouti-related protein (AgRP) is a neuropeptide produced by the AgRP/NPY neurons of the arcuate nucleus in the hypothalamus and it plays a critical role in female puberty and reproduction via an effect on leptin secretion (42). Neonatal MSG injection nearly erased all AgRP immunoreactivity in the hypothalamus (43). Likewise, disappearance of AgRP immunoreactivity in the arcuate nucleus in mice injected with MSG was reported (Table 1) (44).

**Table 1:** Effect of MSG on reproductive neuropeptides


Reproductive neuropeptide	MSG effect	References

VIP	+	(41)
	-	(40)
Neurokinin B	-	(26)
Kisspeptin	+	(24)
POMC	+	(33)
	-	(34)
NPY	+	(30)
	-	(28, 29, 31)
Galanin	-	(37)
Galanin-like peptide	-	(36)
AgRP	-	(43, 44)


MSG; Monosodium glutamate, VIP; Vasoactive intestinal polypeptide, POMC; Proopiomelanocortin, NPY; Neuropeptide Y, AgRP; Agouti-related protein, +; Positive effect of MSG on neuropeptides, and -; Negative effect of MSG on neuropeptides.

### Monosodium glutamate and reproductive neurotrophic factors

Brain-derived neurotrophic factor (BDNF) is a neurotrophic factor thought to be involved in reproductive function because of its widespread expression with receptors located in different parts of the male and female reproductive systems. The results of many studies have indicated its importance in follicles, oocytes, and normal function of spermatozoa (45, 46). A significant downregulation in the hypothalamic levels of BDNF was observed in induced obesity in male mice that received (3 g/kg bw) subcutaneous MSG injections (47). Nerve growth factor (NGF) is a neuropeptide and neurotrophic factor. In addition to its significance in the central and peripheral nervous systems, it has a role in the reproduction system (48). When adult rats were treated with MSG, the expression levels of *Ngf* increased significantly in both the hypothalamus and pituitary, yet remained unchanged in the adrenal gland (30). The immunoreactivity of lowaffinity P75 neurotrophin receptor (p75NR), which is present in the reproductive tract of male rabbits, was reported during sexual maturation (49) p75NR reduced dramatically in the SCN of neonatal rats that received (2 mg/g bw) subcutaneous MSG injections (50).

### Monosodium glutamate and reproductive hormones

MSG causes an imbalance in the secretion of many sex hormones by increasing the secretion of some of these hormones or by decreasing the secretion of others (Table 2).

### Gonadotropin-releasing hormone

GnRH is produced by the hypothalamus, and it has a controlling role on the pituitary gland to secrete the hormones that regulate gonads. Therefore, disorders in the production of this hormone make the production of gametes almost impossible. The mean serum level of GnRH was significantly lower in rats that received oral or subcutaneous MSG compared to the control group of male albino rats (51).

**Table 2:** Effect of MSG on reproductive hormones


Reproductive hormone	MSG effect	References

GnRH	-	(51)
FSH/LH	+	(15)
	-	(52, 53)
Leptin	+	(54-56)
Oxytocin	+	(15, 57)
Progesterone	+	(58)
	-	(59)
Oestrogen	+	(15, 60)
Testosterone	-	(51, 53, 56, 59, 61-65)


MSG; Monosodium glutamate, GnRH; Gonadotropin-releasing hormone, FSH; Folliclestimulating hormone, LH; Luteinizing hormone, +; Sexual hormones positively affected by MSG exposure, and -; Sexual hormones negatively affected by MSG exposure.

In another study, there was considerably less perikarya immunoreactivity in the growth hormone-releasing hormone or LH-releasing hormone (LHRH) neurons of the arcuate nucleus in mice injected with MSG compared to control animals. This was also confirmed by a decrease in size of the anterior lobe of the hypophysis (66).

### Follicle-stimulating hormone/luteinizing hormone

There are many reports that highlighted the negative role of MSG on the production and secretion of folliclestimulating hormone (FSH) and LH; these hormones are critical for the maturation of reproductive organs and the production of male and female gametes. For example, studies have shown that animals injected with MSG have decreased plasma levels of FSH/LH (52, 53). Other studies indicated that the injection or gavage, respectively, of female Sprague Dawley rats with MSG had a negative effect on LH, but not FSH, producing cells in the anterior lobe of the pituitary gland (67, 68). Unlike previous studies that indicated a decrease in the concentration of FSH/LH hormones after oral gavage with MSG, a recent study revealed an increase in anterior pituitary LH and FSH secretion and accompanied by boost secretion of FSHRH and LHRH by paraventricular and supraoptic hypothalamic nuclei, which negatively impacted the reproductive system (15).

### Oestrogen/progesterone

Several studies have reported a negative role of MSG on the production of oestrogens, which negatively affects mammalian metabolic and reproductive function. For example, MSG caused an obvious increase in oestradiol production by follicles (15). Adult female Sprague Dawley rats that received oral MSG treatment had a statistical increase in serum oestrogen levels. This effect decreased after administration of Diltiazem, which prevents the toxic effects of MSG (60). In terms of progesterone, which is considered vital for a stable pregnancy, there are contradictory reports about the effect of MSG on progesterone levels. For example, the results of a study indicated that serum progesterone levels in female pups that received subcutaneous injections of MSG were lower than in the control females (59). The results of other studies indicated that MSG caused an increase in animal hormone production (58). However, in a recent study, the results indicated that there was no considerable effect of MSG on both oestrogen and progesterone production (69).

### Testosterone

Mice with hyperleptinemia from injections of MSG had inhibited secretion of testosterone both *in vivo* and *in vitro* (56). In other studies, subcutaneous injections (4 mg/g bw) of MSG not only reduced the concentration of testosterone in the serum of the pups, but also reduced some of its derivatives such as dihydrotestosterone (59). Oral or subcutaneous administration of MSG to male albino rats considerably reduced serum concentrations of both testosterone and total cholesterol (51). In another study, the consumption of high amounts of MSG by male rats led to a notable decrease in the plasma testosterone levels, which led to partial infertility (53, 65). Moreover, there was an increase in corticosterone and a decrease in testosterone, which causes feminization in the MSGtreated male mice (64). Injections of MSG in neonatal male mice led to disorders in the sexual steroids in general, including testosterone (63). Purified Leydig cells from MSG-injected rats showed *in vitro* concentrations of 17-hydroxyprogesterone, delta-(4)-androstenedione, and testosterone that were significantly lower than control cells (62). Probably the negative effect of MSG was a result of the indirect suppression of hepatic enzymes that were vital to the production of testosterone and other sex steroids like cytochrome P450 2A2 (CYP2A2) and cytochrome P450 3A2 (CYP3A2) (61). However, some studies reported that MSG had no significant effect on serum levels of growth hormone (GH), LH, FSH, cortisol, prolactin, oestradiol, or testosterone (67).

### Oxytocin

When virgin female Charles Foster rats were gavaged with MSG, the force of the uterine contractions increased significantly, which might be due to increased uterus sensitivity to oxytocin (15). In another study, there were increased oxytocin levels in the SCN, arcuate nucleus, and ME, and a decrease in oxytocin level in the paraventricular nucleus in rats that received intraperitoneal injections (4 mg/g bw) of MSG (57).

### Leptin

Adult Siberian hamsters that received subcutaneous injections of MSG in the neonatal stage had higher serum leptin concentrations compared to the control counterparts (55). The same result was obtained in MSG-treated mice (54, 56).

## Conclusion

Most MSG studies were conducted on neonatal animals due to incomplete formation of their blood-brain barriers and MSG cannot cross this barrier. However, MSG is toxic when consumed in large quantities. Subcutaneous or intraperitoneal injections or overconsumption of MSG in adult animals leads to obesity, accompanied by an imbalance in the production of regulatory peptides of the HPG axis, which includes neuropeptides, neurotrophic factors, and hormones that can lead to sexual dysfunction and possibly sterility.

In future studies, we propose to investigate the effect of MSG on the secretion of other hormones and regulating neuropeptides, which have not been studied, with emphasis on oral treatment due to its clinical importance because of MSG consumption as a food flavouring.
